# Sleep Disturbances as a Consequence of Long COVID-19: Insights from Actigraphy and Clinimetric Examinations—An Uncontrolled Prospective Observational Pilot Study

**DOI:** 10.3390/jcm13030839

**Published:** 2024-02-01

**Authors:** Wojciech Tański, Anna Tomasiewicz, Beata Jankowska-Polańska

**Affiliations:** 1 Department of Internal Medicine, 4th Military Clinical Hospital, 50-981 Wroclaw, Poland; dr.wojciech.tanski@gmail.com; 2 Faculty of Medicine, Wrocław University of Science and Technology, 50-376 Wroclaw, Poland; 3 Student Research Club of Surgical Specialties, Wroclaw Medical University, 50-532 Wroclaw, Poland; 4 Center for Research and Innovation, 4th Military Clinical Hospital, 50-981 Wroclaw, Poland

**Keywords:** sleep disturbances, COVID-19, Epworth Sleepiness Scale, Insomnia Severity Index, actigraphy

## Abstract

The COVID-19 pandemic represents a global health and social challenge. However, the impact of a SARS-CoV-2 infection itself on mental health and sleep quality remains poorly understood. The purpose of the present uncontrolled prospective observational pilot study was to evaluate the impact of past COVID-19 disease on the incidence of quantitative and qualitative sleep disturbances. A group of 61 subjects (37 female, 24 male) reported sleep disturbances that had lasted for at least one month and had started after recovery from COVID-19. The study used self-reported instruments: the Epworth Sleepiness Scale (ESS) and Insomnia Severity Index (ISI), as well as an objective method—actigraphy—for quantitative analysis of sleep architecture. It was shown that sleep disturbances most commonly started after recovery (68.3%, n = 41) and lasted for more than one month (83.6%, n = 51). ESS scores of 7.8 ± 5.0 points indicate moderate daytime sleepiness, and ISI scores of 16.3 ± 5.8 points denote moderate insomnia. ESS scores were negatively correlated with total time in bed (r = −0.3780, *p* = 0.003), total sleep time (r = −0.2969, *p* = 0.020), and wakefulness after sleep onset (r = −0.2654, *p* = 0.039). In addition, ESS scores were correlated negatively with the respondents’ age (B = −0.17, *p* = 0.000) and time from COVID-19 onset. A positive correlation was found between wakefulness after sleep onset and ESS scores (B = −0.05, *p* = 0.039). ISI scores were positively correlated with time in bed (r = 0.3275, *p* = 0.010). Female gender was found to be a significant predictor of insomnia’s severity (B = 2.159, t = 3.04, *p* = 0.004). In conclusion, patients with a history of COVID-19 report long-lasting sleep disturbances that do not subside spontaneously. In the patient group studied, moderate levels of daytime sleepiness and insomnia were found. The most frequently reported problems included irregular sleep, frequent awakenings, and difficulty maintaining sleep, while normal sleep duration was preserved. These findings underscore the need for continued attention to the long-term consequences of COVID-19 on sleep health and emphasize the importance of targeted interventions to address these enduring sleep disturbances in affected individuals.

## 1. Introduction

COVID-19 is a disease caused by an infection with SARS-CoV-2, a virus in the same family as the previously known SARS-CoV and MERS-CoV. The first cases of COVID-19 were recorded in December 2019 in Wuhan, China, and in March 2020 the WHO declared the disease a pandemic [1]. The most common COVID-19 symptoms include fever, cough, weakness, poor appetite, shortness of breath, headaches and muscle aches, nausea, vomiting, and olfactory dysfunction [2]. The course of the disease varies from asymptomatic, through forms manifesting with mild (40%) or moderate (40%) symptoms, to severe disease (15%) requiring oxygen therapy or critical cases (5%) leading to severe acute respiratory syndrome, septicemia or septic shock, and multiple organ failure, including acute kidney failure [3].

An association has been found between a more severe course of the disease, on the one hand, and older age, smoking, immune suppression, and a number of chronic diseases, such as hypertension, diabetes, cardiovascular and respiratory diseases, and cancer, on the other [2]. In addition to COVID-19 itself, there is ongoing interest in what is called “long COVID”, where symptoms associated with the SARS-CoV-2 infection that appear about 3 months after the onset of the disease persist for over 2 months. Frequently reported long COVID symptoms include fatigue, muscle aches, palpitations, cognitive impairment, shortness of breath, anxiety, chest pain, and joint pain [4]. It was reported that 71% of patients experienced the onset of long COVID, while the remaining 28% achieved complete symptom remission 3 months after acute infection [5]. The post-COVID-19 phase is also strongly associated with mood disorders [6].

Sleep disturbances are a group of disorders that interfere with normal sleep patterns. In the ICSD-3, they are grouped under six major categories: insomnia, sleep-related breathing disorders, hypersomnolence and narcolepsy, circadian rhythm disorders, sleep-related movement disorders, and parasomnias [7]. Sleep can be evaluated using subjective instruments, e.g., the Epworth Sleepiness Scale (ESS) and the Insomnia Severity Index (ISI), and using objective approaches, such as actigraphy. 

Interest in COVID-19-related sleep disturbances emerged early in the pandemic. A 2022 meta-analysis of 63 papers showed that sleep disorders were present in approx. 24% of patients who had been ill with COVID-19 between 3 and 6 months prior to the study, 29% in the case of 6–9 months, and 30% of patients who had been ill more than 12 months prior to the study. However, the numbers reported in particular studies varied considerably [8]. Despite the upward trend identified in the meta-analysis with regard to the prevalence of sleep disorders as more time passed from the infection, some papers have reported the opposite tendency [9]. Published studies either confirm [10] or deny [11] an association between disease severity and the risk of developing a sleep disorder. Female gender has also been suggested as a risk factor for COVID-19-associated sleep disturbances [12].

Sleep is an integral part of each day in a human’s life. Sleep deficits may lead to a number of disturbances in physical, psychological, and social functioning. Insufficient sleep has been linked to glucose tolerance disorders, high blood pressure, sympathetic nervous system activation, increased blood cortisol levels, and decreased leptin levels; insufficient sleep has also been described as a factor favoring obesity, due to the related increase in appetite. A cohort study also showed an association between short sleep (5 or fewer hours per day) and an increased risk of ischemic heart disease and diabetes [13].

Furthermore, sleep disturbances have an impact on mental health, as they have been linked to a higher risk of depression and anxiety, increased irritability, and low mood. Associations have also been described between a variety of sleep disturbances and poorer quality of life (QoL); impaired concentration, alertness, memory, and learning; and reduced work performance [14]. In the elderly population, sleep disturbances may be a risk factor for dementia [15]. Considering the prevalence of sleep disturbances and the broad range of potential consequences, understanding the potential contributors to these disorders seems crucial. Such investigations could lead to the development of adequate prevention methods and a system for early detection of emerging abnormalities, opening the way for rapid intervention and treatment.

The primary objective of the present paper is to demonstrate the impact of a past COVID-19 infection on sleep disturbances, both with the use of self-reported measures (ESS and ISI), and with an objective method (actigraphy). Its secondary objective is to assess the impact of factors such as gender, age, and actigraphy measurements on the self-reported sleep parameters. 

## 2. Materials and Methods

### 2.1. Study Design

This uncontrolled prospective observational pilot study was conducted at the Department of Internal Medicine and Clinical Research Support Center at the 4th Military Clinical Hospital (Poland) between 1 June 2022 and 31 December 2023. The study protocol was approved by the Bioethics Committee of the Military Medical Chamber in Warsaw (approval no.: 216/2022, approval date: 22 April 2022), and the study was conducted in accordance with the ethical principles of the Declaration of Helsinki. All patients provided written informed consent to participate in the study.

### 2.2. Enrollment

The study included patients who had had COVID-19 and were suffering from sleep disturbances (i.e., subjective sleep abnormalities persisting for a minimum of one month, which had appeared after a COVID-19 infection, and which the patient associated with the disease). The past COVID-19 infection had been confirmed by a laboratory test (PCR or antigen) at least 3 months before enrollment. Other inclusion criteria included sufficient performance status to participate in the study (i.e., ECOG grade 0/1/2), sufficient cognitive state (i.e., >24 points in the MoCA scale), and written informed consent to participate. 

Enrollment was performed via the hospital’s website. An information brochure was also created and handed out to patients, and information posters were displayed in cooperating healthcare institutions. Information about the study was also broadcast by regional radio and television stations as part of their health-themed programming.

### 2.3. Study Protocol

Patients applied to participate in the study themselves by calling a dedicated telephone number or reporting in person to a study center. At the first stage, the study coordinator conducted a standardized interview to confirm a past COVID-19 infection and the presence of complaints (i.e., sleep disturbances) that the patients associated with the infection. Patients included in the study completed an Epworth Sleepiness Scale (ESS) questionnaire for sleep disorder diagnosis. During a visit, the patients underwent a physical examination, had their history noted by a nurse, and were assessed for muscle strength (with a dynamometer), anthropometric parameters (i.e., body weight, height, and BMI), and primary vital signs (i.e., blood pressure, heart rate, SpO_2_). Later, each patient was seen by a physician, who took down their detailed history, including their history of COVID-19, other chronic diseases, and medication taken. Then, the patient received an actigraph unit, enabling a quantitative assessment of sleep architecture, with specific instructions to use the unit for the following 7–10 days so as to have their daytime and nighttime activity recorded with a special sensor. During the second visit, between 7 and 10 days after being fitted with the actigraph unit, a psychiatrist analyzed the patient’s measurement results. During a consultation with the patient, any problems identified were analyzed and discussed, and proper management to reduce the disturbances was recommended. Patients requiring further diagnostics were referred for polysomnography and specialist treatment.

### 2.4. Outcomes

The ESS is a self-reported scale used in diagnostics for sleep disturbances such as obstructive sleep apnea, where the patient scores (on a scale of 0–3) their likelihood of becoming sleepy in specific daily situations [16].

The ISI is used to assess sleep abnormalities. The questionnaire comprises seven items concerning the severity of problems associated with falling asleep, staying asleep, and waking up too early. It also evaluates satisfaction with the current quality of sleep, difficulties in daily functioning, perceived noticeability of the problems to others, and the patient’s distress caused by the disorder. Answers are provided on a 5-item Likert scale. Scores above 15 indicate clinically significant insomnia [17].

The present study also included objective assessment using actigraphy. An actigraph unit is a medical device in the form of a wristband that monitors the patient’s vital signs. Patients in the study were evaluated with this device for 10 days. This method is used to monitor sleep quality and circadian rhythm stability. The system records the patient’s daily physical activity, including the number of steps taken, energy expenditure, and periods of sleep. It enables the collection and analysis of data on physical activity and sleep parameters, with accurate, 24-hour recording of daily sleep and physical activity, including latency (min), efficiency (%), total time in bed (min), total sleep time (TST) (min), wakefulness after sleep onset (WASO) (min), number of awakenings, average awakening (min), number of sleep episodes, and study duration (days) [18].

### 2.5. Statistical Analysis

Statistical analysis was performed using Statistica 13 software (TIBCO Software Inc., Palo Alto, CA, USA). For continuous variables, arithmetic means, median values, standard deviations, and ranges of variation (extreme values) were calculated. For qualitative variables, the frequency of their occurrence was calculated (as a percentage). All of the quantitative variables tested were then analyzed with the Shapiro–Wilk test to determine the type of distribution. Dependence between the selected alternatives was determined using Spearman’s rank correlation test. An alpha level of 0.05 was used in all comparisons. In addition, the impact of selected factors on sleep quality was evaluated using linear regression (with a single-factor model of predictors included in the analysis). Non-standardized and standardized regression factors, standard errors, and statistical significance were calculated. In the next step, a multifactor model was built (using the progressive stepwise method), including variables that had a *p*-value of 0.30 or lower in the single-factor model.

## 3. Results

The study included 61 patients, 60.7% (n = 37) female and 39.3% (n = 24) male. Table 1 shows their detailed demographic characteristics, including the patients’ age, height, weight, gender, education level, place of residence, and living situation.

Table 2 contains data on COVID-19 in the patient group. The mean time from disease onset was 491.9 days (min–max: 136.0–1096.0 days; SD = 249.4 days), the mean duration of the acute disease phase was 7.9 days (min–max: 0.0–42.0 days; SD = 8.2 days), and the mean number of vaccine doses received was 1.5 (min–max: 0.0–4.0 doses; SD = 1.4 doses).

Infection was typically confirmed by PCR (51.7%, n = 30), as part of diagnostics performed in a primary care context (49.2%, n = 29). In most of the patients, the disease had a mild course (83%, n = 50), and symptomatic treatment was most commonly administered (93.4%, n = 57). Symptoms that started within 3 months after the onset of COVID-19 and persisted for at least 2 months mainly included poorer exercise tolerance (62.3%, n = 38) and fatigue (62.3%, n = 38). Notably, cognitive function and memory impairment, commonly referred to as “brain fog”, were reported by 52.5% (n = 32) of patients, while mood disorders, including anxiety and even depressive symptoms, were reported by 44.3% (n = 27) (Table 3).

Table 4 contains data on sleep in the patient group. Sleep disturbances most commonly started after recovery (67.2%, n = 41) and lasted for more than one month (83.6%, n = 51). The most commonly used stimulant was coffee (54.1%, n = 33). Most respondents had a regular sleep schedule (62.3%, n = 38). The most reported behavior affecting sleep was using a phone or other electronic devices up to an hour before going to sleep (65%, n = 39).

Table 5 contains the patients’ questionnaire and actigraphy data. The mean study duration was 9.9 days (min–max: 6.0–12.0 days; SD = 1.2 days) (Table 5).

Correlation analysis was performed for ESS and ISI scores and a number of variables related to sleep quality (Table 6). Total time in bed was negatively correlated with ESS scores (r = −0.3780, *p* = 0.003), which means that more time spent in bed was associated with lower scores on the sleepiness scale. Total sleep time was negatively correlated with ESS scores (r = −0.2969, *p* = 0.020), which suggests that longer sleep is associated with less sleepiness. Wakefulness after sleep onset (WASO) was negatively correlated with ESS scores (r = −0.2654, *p* = 0.039), which indicates that longer periods of wakefulness after falling asleep are associated with worse sleepiness. The correlation between the ISI score and total time in bed was 0.3275, and this finding was statistically significant (*p* = 0.010). This means that there was a positive association between the ISI score and the time spent in bed. Higher ISI scores (which indicate greater severity of insomnia) are linked to more time spent in bed, suggesting that people with more severe insomnia may try to compensate for their difficulty falling and staying asleep by spending a longer time in bed.

Linear regression analysis was performed to understand the impact of various variables on the severity of insomnia, as measured by the ISI (Table 7). The regression coefficient was higher for women (B = 2.159) than for men, which means that gender is a statistically significant predictor of insomnia’s severity (t = 3.04, *p* = 0.004). Moreover, the independent variable “Total time in bed (min)” had a statistically significant impact on the dependent variable “Insomnia Severity Index” (β = 0.33, t = 2.66, *p* = 0.010). The regression coefficient (β) was 0.33, which means that every additional minute spent in bed contributed to a 0.33-point increase on the ISI.

Other variables, including age, days since disease onset, disease course, duration of acute COVID-19, sleep latency, sleep efficiency, total time in bed, total sleep time (TST), time of wakefulness after sleep onset (WASO), number of awakenings, mean duration of awakening, and number of sleep episodes, did not produce statistically significant effects in terms of insomnia severity. This suggests that more time spent in bed may be associated with greater insomnia severity, which has possible significant clinical or psychological implications.

Another linear regression analysis revealed a number of statistically significant associations between the variables studied and the ESS scores (Table 8). A significant negative correlation was found between age and ESS scores (B = −0.17, t = −3.70, *p* = 0.000, ß = −0.43), i.e., older respondents had lower sleepiness scores, which may indicate less sleepiness in older individuals. A significant negative correlation was found between the time since COVID-19 onset and ESS scores (B = −0.01, t = −2.27, *p* = 0.027, ß = −0.29). This means that longer time since COVID-19 was associated with lower sleepiness scores, suggesting that long-term complications of the disease may affect sleepiness. In addition, a significant negative correlation was found between time in bed and ESS scores (B = −0.03, t = −3.14, *p* = 0.003, ß = −0.38). This indicates that the sleepiness scores decrease as the time spent in bed increases, possibly suggesting better sleep quality. A significant negative correlation was also found between total sleep time and ESS scores (B = −0.03, t = –2.39, *p* = 0.020, ß = −0.30). This means that longer duration of sleep was associated with lower sleepiness scores, suggesting that longer sleep entails better sleep quality. A significant positive correlation was observed between wakefulness after sleep onset and ESS scores (B = −0.05, t = −2.11, *p* = 0.039, ß = −0.27). This means that sleepiness scores increase as the time spent awake after falling asleep increases, possibly suggesting poorer sleep quality. These findings collectively indicate that ESS scores are significantly influenced by various factors, including age, the duration since the onset of COVID-19, time spent in bed, total sleep time, and the duration of wakefulness after sleep onset.

## 4. Discussion

As regards recent scientific reports on sleep quality in patients with a history of COVID-19, there have been numerous studies evaluating sleep in different time periods to understand the development of this clinical disorder after COVID-19, along with its long-term consequences [19]. Ongoing research focuses on identifying any permanent changes in sleep quality in people with a history of COVID-19, and on determining the impact(s) of any such changes on the overall health and QoL of patients after recovery. This is important for a better understanding of the long-term effects of COVID-19, and for the development of healthcare management strategies for those with a history of the disease.

The systematic review and meta-analysis on sleep disorders during the COVID-19 pandemic by Jahrami et al. [20] showed that the global prevalence of these problems was around 40.49%. Six major groups were identified. The estimated prevalence of sleep problems was 52.39% in patients infected with COVID-19, 45.96% in children and adolescents, 42.47% in healthcare professionals, 41.50% in populations requiring healthcare, 41.16% in higher-education students, and 36.73% in the general population. Sleep disturbances were more prevalent during lockdown periods (42.49%) compared to times with no lockdown in place (37.97%). The authors reported that four out of ten people experienced sleep problems during the pandemic, the most affected groups being COVID-19 patients, children, and adolescents.

In a British population-based study, Kantor et al. [21] reported that a total of 68.9% of respondents met the criteria for poor sleep quality. The odds of poor sleep quality increased along with attitudes related to the COVID-19 pandemic, such as higher levels of stress, fear, or loneliness, in a dose-dependent manner. The link between poor sleep quality and attitudes towards the pandemic points to possible interventions in the fields of public health and sleep medicine, highlighting the need for continued research within the specialty.

The subject of the present prospective observational pilot study on the impact of past COVID-19 disease on the incidence of objective and subjective sleep disturbances is important and highly topical, particularly in light of the fact that, despite the end of the pandemic, COVID-19 is still present in the population [22], and the disease may have a long-lasting impact on health due to “long COVID” symptoms [23].

Our study identified an alarmingly high prevalence of sleep disturbances; 67% of participants reported sleep disturbances after recovering from COVID-19, demonstrating that this experience is common. As many as 84% of the patients reported disturbances lasting for more than one month, which shows that the problems tend to be persistent and do not subside on their own. This, in turn, may have a catastrophic impact on these patients’ QoL and overall health. Our findings are consistent with the published data, with the reported prevalence of sleep disorders ranging from 6% to more than 70% [24]. In an online international study on post-COVID-19 conditions, 78.58% of respondents experienced sleep disorders, including insomnia, sleep-related breathing disorders, circadian rhythm disorders, and sleep-related movement disorders [25]. 

In terms of questionnaire scores, we recorded a mean ESS score of 7.8 points (SD = 5.0), indicating moderate daytime sleepiness in the group studied. The ESS is scored between 0 and 24 points, with higher scores indicating more daytime sleepiness, and results below 10 indicate a normal overall daytime sleepiness level [26]. The mean ISI score in the group, 16.3 points (SD = 5.8), indicates a moderate severity of insomnia in the population studied. ISI scores range between 0 and 28 points, with higher scores indicating more severe insomnia. In our study, the mean fell in the 15–21-point range, classified as moderately severe insomnia.

In their study on COVID-19-related sleep disorders in patients with obstructive sleep apnea (OSA), Labarca et al. [27] also used the ESS and ISI, as well as actigraphy measurements performed over 7 days. There was no difference between the OSA and non-OSA groups (9.56 vs. 7.5 points).

Studies to date confirm that the problem of COVID-19-related sleep disorders is significant and widespread globally. Research shows that many people experienced difficulty falling or staying asleep during the pandemic, with a possible negative impact on their overall health and QoL. Findings from Korea [28] suggest that one in three adults (32.9%) suffers from insomnia, and 25.6% and 22.7% of respondents reported difficulty falling and staying asleep, respectively, at least two nights a week. A study by Irdissi et al. [29] revealed a high prevalence of sleep disorders, especially insomnia (56.0%) and daytime sleepiness (9.9%). In addition, 29.5% of respondents experienced anxiety, and 35.6% had depression symptoms. The authors’ analysis demonstrated that insomnia was significantly dependent on the respondents’ residence and history of chronic disease: those who lived in urban areas and were chronically ill had higher scores, which was not observed in our study.

Ramos et al. [30] confirmed that the COVID-19 pandemic had a significant impact on sleep disorders in the elderly, bringing about an increase in chronic insomnia and generalized anxiety disorders. In terms of sleep disturbances, the prevalence of chronic insomnia increased by 46%. No significant differences were found in the case of other types of sleep disorders, such as obstructive sleep apnea (92%), restless leg syndrome (24%), periodic limb movement disorders (32%), REM sleep behavior disorders (8%), and circadian rhythm disorders (4%). Polysomnography revealed significant differences in terms of sleep stages, but not in other sleep architecture parameters, such as sleep latency, REM sleep latency, total sleep time, shares of all sleep stages (N1, N2, N3, and REM), wakefulness after sleep onset, arousal rate, periodic leg movements (PLM) during sleep, or the apnea–hypopnea index (AHI). More than one-half of the patients (54%) experienced moderate or severe clinical insomnia. Significant increases in state anxiety, trait anxiety, and sleepiness were also observed in the study subjects.

The literature also includes a number of studies on the use of actigraphy in COVID-19-related sleep disorders. The latest systematic review by Ferreira-Souza et al. [31] analyzed 15 studies where sleep quality during the COVID-19 pandemic, as measured by actigraphy, was reported as “poor”. The authors concluded that actigraphy may be an important tool for evaluating individual circadian rhythms and providing recommendations during an ongoing pandemic. Moreover, since actigraphy provides objective data for sleep evaluation, its inclusion as an integral part of sleep hygiene strategies has been suggested.

In our study, actigraphy showed a mean latency (time required to fall asleep) of 6.1 min, which indicates normal sleep latency, considering that a mean latency under 5 min is a sign of pathological sleepiness, and in the absence of excessive sleepiness, the latency is above 10 min. Sleep efficiency of 86.1% means that the patients slept for about 86.1% of their time in bed. This suggests that they stayed awake for some of the time or remained in bed after awakening. The participants spent an average of 475.3 min (or approx. 7.9 h) in bed, which was longer than their mean sleep time and may suggest irregular sleep. Their mean total sleep time was 408.4 min (approx. 6.8 h), indicating a fairly normal sleep duration. However, the mean time of the first awakening after sleep onset (61.0 min) and the number of awakenings (16.6) may suggest that the participants had difficulties maintaining sleep. In turn, the mean duration of each awakening (4.4 min) suggests that the awakenings were relatively short and that the participants had no trouble falling asleep again.

Furthermore, higher sleepiness scores on the ESS were significantly correlated with shorter time in bed, shorter total sleep time, and faster awakening after falling asleep. Higher insomnia scores on the ISI scale were significantly correlated with longer time in bed. Moreover, the following factors were identified as significant predictors of better ESS scores: older age, longer time since COVID-19, longer time in bed, longer sleep time, and shorter time of wakefulness after sleep onset. Significant predictors of insomnia on the ISI scale include female gender and longer time in bed. Jeon et al. [28] used multiple logistic regression analysis to confirm a correlation between insomnia and female gender; additionally, associations were identified with nighttime work and marital status (single). This is contrary to findings from a meta-analysis by AlRasheed et al. [32], who observed no apparent association of insomnia symptoms, as measured by the ISI, with gender or age. Overall, an estimated 52.57% of the population experienced symptoms of insomnia, either mild or clinically significant. Approximately 16.66% of the population suffered from clinically significant insomnia, including 13.75% with moderate insomnia, and 2.50% with severe insomnia.

Interestingly, coffee was the main stimulant used by most of the patients in our study (54%). The caffeine in coffee may affect sleep, especially when consumed in large quantities or late during the day. It may independently contribute to sleep disturbances in some individuals [31]. Still, 62% of patients had a regular sleep schedule, which is clearly positive, since regular sleep and waking up at a fixed time are significant to the maintenance of a healthy circadian rhythm and good sleep quality [33]. Notably, a large number of the respondents in our study (65%) reported using a phone or other electronic devices up to an hour before going to sleep. This is important, as the blue light emitted by electronic device screens may interfere with falling asleep by dysregulating the circadian rhythm [34]. Therefore, this may be another independent factor preventing falling asleep, leading to poorer sleep quality.

The relationship between COVID-19 incidence and previous sleep quality should also be noted. A recent study by Quan et al. [35] found that both insomnia and poor sleep quality were associated with a higher risk of COVID-19 infection. The association was stronger for poor sleep quality than for insomnia, and the former was also linked to a higher risk of COVID-19-related hospitalization. Vargas et al. [36] confirmed that insomnia may be associated with longer-lasting chronic COVID-19 symptoms. Patients suffering from insomnia at the beginning of the study (62.8%) were more likely to undergo COVID-19 testing than those without insomnia (57.4%). This clearly confirms the role of adequate sleep quality in the context of COVID-19, both for reducing the risk of hospitalization in the course of the SARS-CoV-2 infection itself, and for preventing quantitative and qualitative sleep disorders after recovery from COVID-19.

### 4.1. Study Limitations

The present study has certain methodological limitations that merit discussion. One weakness of this study is its single-center design, which prevents generalization of our findings to the entire Polish population or to the cultural and systemic contexts of other countries. Objective assessment of sleep disturbances was performed using actigraph units to monitor sleep parameters. Using more precise, specialized methods for sleep disorder diagnosis, such as polysomnography, should be considered in future studies. However, in our study, actigraphy functioned primarily as a screening test for the assessment of sleep disturbances. Actigraphy served the crucial purpose of providing an initial, broad overview of sleep parameters, allowing for the identification of potential sleep disturbances among the participants. However, it is important to acknowledge that, as a screening tool, actigraphy has inherent limitations in terms of its ability to provide in-depth diagnostic information compared to more specialized methods like polysomnography. While actigraphy offers valuable insights into sleep patterns, future research endeavors may consider complementing its use with additional diagnostic measures to enhance the precision and comprehensiveness of sleep disorder assessments in individuals with a history of COVID-19.

Moreover, adding more self-reported and standardized instruments, such as the Pittsburgh Sleep Quality Index (PSQI), the Athens Insomnia Scale (AIS), or the STOP-BANG Questionnaire on sleep apnea, could also be suggested. The reliability of the findings could be improved by including a control group of people who had not been ill with COVID-19, but who suffer from sleep disturbances and are similar to the study group in terms of other characteristics, such as age, gender, and overall health. Another important aspect is determining the duration of follow-up after recovery from COVID-19, which would help understand whether the sleep disturbances are short-lived or persistent in the long term. Importantly, future studies should consider the potential risk factors for sleep disturbances in patients with a history of COVID-19, such as disease severity, out-of-hospital symptoms, comorbidities, or current pharmaceutical treatment. An assessment of patients’ mental health should also be included, as sleep disturbances are often associated with such mental problems as anxiety or depression, and being ill with COVID-19 itself can affect patients’ mental state.

### 4.2. Practical Implications

Our study may contribute to a better understanding of the impact of COVID-19 on sleep quality and help suggest strategies for patients who have recovered from the disease but suffer its long-term consequences. One notable feature of our study is the use of a variety of methods for sleep assessment, both subjective (i.e., self-reported sleep quality questionnaires) and objective (i.e., actigraphy measurements of various quantitative sleep parameters). This approach allowed for a comprehensive assessment of the contribution of COVID-19 to the development of any sleep abnormalities. The actigraphy results indicated some sleep disturbances in patients with a history of COVID-19, such as short sleep, frequent awakenings, and/or difficulty maintaining sleep. The analysis of these parameters may improve the understanding of how COVID-19 affects sleep’s quality and architecture in patients, as well as identifying potential therapeutic needs in terms of improving sleep quality and eliminating the adverse consequences of poor sleep. Actigraphy can be an effective measurement tool in the analysis of individual day and night rhythms, helping to provide recommendations regarding the long-term health consequences of COVID-19 by estimating sleep latency, total sleep time, wakefulness after sleep onset, and sleep efficiency. Providing knowledge on COVID-19-related sleep disturbances may improve the understanding of the long-term effects of the disease in the context of general health and wellbeing. It can also provide guidance on patient management to improve sleep’s quality and duration. The findings reported here may be useful in the design of further cohort or epidemiological studies, as well as prospective studies and controlled trials concerning the impact of COVID-19 on the mental and physical health of the population. They may also help in understanding the impact of the long-term consequences of the disease on patient QoL. Our findings may be a starting point for further investigations into the mechanisms and causes of sleep disturbances in patients with a history of COVID-19.

## 5. Conclusions

Patients with a history of COVID-19 report long-lasting sleep disturbances that do not subside spontaneously. After the observation period, moderate levels of daytime sleepiness and insomnia were found in the patient group studied. The most frequently reported problems included irregular sleep, frequent awakenings, and difficulty maintaining sleep, while normal sleep duration was preserved. Shorter time in bed, shorter total sleep time, and faster awakening after sleep onset were found to be associated with more sleepiness. Longer time in bed was associated with more severe insomnia. Furthermore, older age, longer time since COVID-19, longer time in bed, longer duration of sleep, and shorter time of wakefulness after sleep onset were predictors of lower sleepiness levels, while female gender and longer time in bed were predictors of insomnia.

## Figures and Tables

**Table 1:** Demographic characteristics of the study participants (n = 61).

**Group n = 61**							
**Variable**	**x**	**Me**	**Min**	**Max**	**Q1**	**Q3**	**SD**
Age (years)	52.4	50.0	19.0	78.0	45.0	62.0	12.7
Height (cm)	170.0	170.0	152.0	191.0	164.0	175.0	9.1
Weight (kg)	76.8	72.0	50.0	141.0	63.0	85.0	18.8
**Variable**	**Category**			**N**		**%**	
Gender	Female			37		60.7	
	Male			24		39.3	
Education	Primary			1		1.7	
	Vocational			2		3.3	
	High school			16		26.7	
	College/university			41		68.3	
Residence	Rural			4		6.7	
	Urban < 20,000 residents			2		3.3	
	Urban < 100,000 residents			10		16.7	
	Urban > 100,000 residents			44		73.3	
Living situation	Alone			9		15.0	
	With a partner			8		13.3	
	With family			43		71.7	

Abbreviations: x, mean; Me, median; Q1, lower quartile; Q3, upper quartile; Min, minimum value; Max, maximum value; SD, standard deviation; n, number of respondents; %, percentage of respondents.

**Table 2:** Biological characteristics of the study participants (n = 61).

Group n = 61							
Variable	x	Me	Min	Max	Q1	Q3	SD
Heart rate [bpm] (n = 61)	73.9	74.0	48.0	111.0	69.0	80.0	11.0
SBP [mmHg] (n = 61)	123.4	122.0	100.0	149.0	115.0	130.0	10.8
DBP [mmHg] (n = 61)	77.6	80.0	60.0	98.0	73.0	80.0	7.4

Abbreviations: x, mean; Me, median; Q1, lower quartile; Q3, upper quartile; Min, minimum value; Max, maximum value; SD, standard deviation; n, number of respondents; %, percentage of respondents; SBP, systolic blood pressure; DBP, diastolic blood pressure.

**Table 3:** History of COVID-19.

**Group n = 61**								
**Variable**		**x**	**Me**	**Min**	**Max**	**Q1**	**Q3**	**SD**
When did you become ill with COVID? [number of days since disease onset] (n = 57)		491.9	396.0	136.0	1096.0	306.0	761.0	249.4
Duration of the acute phase of the disease in days (n = 60)		7.9	5.0	0.0	42.0	3.0	10.5	8.2
Number of vaccine doses received prior to onset of the disease (n = 61)		1.5	2.0	0.0	4.0	0.0	3.0	1.4
**Variable**	**Category**	**N**			**%**			
Manner of confirming the infection (n = 58)	Antigen test	4			6.9			
	PCR	30			51.7			
	PCR and antigen test	8			13.9			
	Other	2			3.4			
	No confirmation	14			24.1			
Context of diagnosis (n = 59)	Primary care	29			49.2			
	Emergency department	0			0.0			
	Hospital	7			11.9			
	Other	23			39.0			
Disease course (n = 60)	Mild	50			83.3			
	Moderate	7			11.7			
	Severe	3			5.0			
COVID treatment administered (n = 61)	Symptomatic	57			93.4			
	Steroid therapy	8			13.1			
	Low-molecular-weight heparin	4			6.6			
	Antiviral drugs	4			6.6			
Symptoms that appeared within 3 months of COVID-19’s onset and persisted for at least 2 months (n = 61)	Fatigue	38			62.3			
	Cognitive impairment, “brain fog”, memory impairment	32			52.5			
	Reduced exercise tolerance	38			62.3			
	Difficulty breathing, shortness of breath, cough	25			41.0			
	Chest pain, palpitations, tachycardia	21			34.4			
	Mood disorders, anxiety, depression	27			44.3			
	Dizziness and headaches	15			24.6			
	Muscle and joint pain	26			42.6			
	Abdominal pain, gastrointestinal discomfort	7			11.5			
	Olfactory dysfunction	14			23.0			

Abbreviations: x, mean; Me, median; Q1, lower quartile; Q3, upper quartile; Min, minimum value; Max, maximum value; SD, standard deviation; n, number of respondents; %, percentage of respondents; PCR, polymerase chain reaction.

**Table 4:** Questions about sleep disturbances.

Group n = 61			
Variable	Category	n	%
First occurrence of sleep disturbances	During illness	20	32.8
	After recovery	41	67.2
Duration of sleep disturbances	A few days	7	11.5
	2–3 weeks	3	4.9
	More than 1 month	51	83.6
Stimulants used	Coffee	33	54.1
	Tea	21	34.4
	Alcohol	4	6.6
	Nicotine	7	11.5
Sleep schedule	Irregular	23	37.7
	Regular	38	62.3
Use of phone or other electronic devices up to an hour before going to sleep	No	21	35.0
	Yes	39	65.0
Activities in the bedroom other than sleep or sex (e.g., watching TV, reading in bed)	No	38	63.3
	Yes	22	36.7
Exercise 0.5–3 h before bedtime	No	39	65.0
	Yes	21	35.0
Past use of sleeping pills	No	46	75.4
	Yes	15	24.6
Current use of sleeping pills	No	48	78.7
	Yes	13	21.3

Abbreviations: N, number of respondents, %, percentage of respondents.

**Table 5:** Questionnaire scores and actigraphy results.

Group n = 61							
Variable	x	Me	Min	Max	Q1	Q3	SD
ESS (score)	7.8	8.0	0.0	19.0	4.0	12.0	5.0
ISI (score)	16.3	18.0	0.0	27.0	13.0	20.0	5.8
**Actigraphy**							
Latency (min)	6.1	5.2	0.1	19.7	3.1	8.1	4.3
Efficiency (%)	86.1	86.5	73.5	94.9	83.1	89.5	4.9
Total time in bed (min)	475.3	466.7	370.0	616.7	445.7	507.8	55.1
Total sleep time (TST) (min)	408.4	411.0	321.4	512.0	379.2	439.2	47.5
Wakefulness after sleep onset (WASO) (min)	61.0	58.6	22.0	132.9	40.1	74.9	26.4
Number of awakenings	16.6	16.0	8.0	26.0	11.9	20.6	5.0
Average awakening (min)	4.4	3.4	2.0	26.0	2.9	4.3	4.2
Number of sleep episodes	8.9	9.0	4.4	11.0	9.0	10.0	1.4
Study duration (days)	9.9	10.0	6.0	12.0	10.0	10.0	1.2

Abbreviations: x, mean; Me, median; Q1, lower quartile; Q3, upper quartile; Min, minimum value; Max, maximum value; SD, standard deviation; ESS, Epworth Sleepiness Scale; ISI, Insomnia Severity Index.

**Table 6:** Analysis of correlations between Epworth Sleepiness Scale and Insomnia Severity Index scores and objective actigraphy readings.

Actigraphy Parameter	ESS	ISI
Latency (min)	0.0757	0.1072
	*p* = 0.562	*p* = 0.411
Efficiency (%)	0.1677	−0.1436
	*p* = 0.196	*p* = 0.270
Total time in bed (min)	−0.3780	0.3275
	*p* = 0.003	*p* = 0.010
Total sleep time (TST) (min)	−0.2969	0.2466
	*p* = 0.020	*p* = 0.055
Wakefulness after sleep onset (WASO) (min) (n = 61)	−0.2654	0.2239
	*p* = 0.039	*p* = 0.083
Number of awakenings	–0.1366	0.2438
	*p* = 0.294	*p* = 0.058
Average awakening (min)	–0.1229	0.1084
	*p* = 0.345	*p* = 0.406
Number of sleep episodes	−0.0525	−0.0316
	*p* = 0.688	*p* = 0.809

Abbreviations: ESS, Epworth Sleepiness Scale; ISI, Insomnia Severity Index.

**Table 7:** Linear regression analysis for the impact of selected variables on the Insomnia Severity Index.

Variable		ISI				
		B	SE	t	*p*-Value	ß
Age		−0.003	0.06	−0.06	0.954	−0.01
Gender	Male	Ref.				
	Female	2.159	0.71	3.04	0.004	0.37
When did you become ill with COVID-19? [number of days since disease onset]		0.004	0.00	1.15	0.257	0.15
Course of COVID-19	Mild	Ref.				
	Moderate	1.059	1.86	0.57	0.572	0.12
	Severe	−1.846	2.37	−0.78	0.439	−0.17
Duration of the acute phase of COVID-19 (days)		0.125	0.08	1.47	0.146	0.19
Latency (min)		0.145	0.17	0.83	0.411	0.11
Efficiency (%)		-0.168	0.15	−1.11	0.270	−0.14
Total time in bed (min)		0.034	0.01	2.66	0.010	0.33
Total sleep time (TST) (min)		0.030	0.02	1.95	0.055	0.25
Wakefulness after sleep onset (WASO) (min) (n = 61)		0.049	0.03	1.76	0.083	0.22
Number of awakenings		0.280	0.15	1.93	0.058	0.24
Average awakening (min)		0.150	0.18	0.84	0.406	0.11
Number of sleep episodes		−0.132	0.54	−0.24	0.809	−0.03

Abbreviations: B, non-standardized regression coefficient B; SE, standard error; t, B/standard error; ß, standardized regression coefficient ß; ISI, Insomnia Severity Index.

**Table 8:** Linear regression analysis for the impact of selected variables on the Epworth Sleepiness Scale.

Variable		ESS				
		B	SE	t	*p*-Value	ß
Age		−0.17	0.05	−3.70	0.000	−0.43
Gender	Male	Ref.				
	Female	−0.62	0.65	−0.95	0.348	−0.12
When did you become ill with COVID-19? [number of days since disease onset]		−0.01	0.00	−2.27	0.027	−0.29
Course of COVID-19	Mild	Ref.				
	Moderate	1.32	1.61	0.82	0.416	0.18
	Severe	−0.40	2.04	−0.19	0.846	−0.04
Duration of the acute phase of COVID-19 (days)		−0.10	0.08	−1.27	0.210	−0.16
Latency (min)		0.09	0.15	0.58	0.562	0.08
Efficiency (%)		0.17	0.13	1.31	0.196	0.17
Total time in bed (min)		−0.03	0.01	−3.14	0.003	−0.38
Total sleep time (TST) (min)		−0.03	0.01	−2.39	0.020	−0.30
Wakefulness after sleep onset (WASO) (min) (n = 61)		−0.05	0.02	−2.11	0.039	−0.27
Number of awakenings		−0.13	0.13	−1.06	0.294	−0.14
Average awakening (min)		−0.15	0.15	−0.95	0.345	−0.12
Number of sleep episodes		−0.19	0.47	−0.40	0.688	−0.05

Abbreviations: B, non-standardized regression coefficient B; SE, standard error; t, B/standard error; ß, standardized regression coefficient ß; ESS, Epworth Sleepiness Scale.

## Data Availability

The authors confirm that all data underlying the findings described in this manuscript are fully available to all interested researchers upon request.
